# Effect of an Intraorifice Barrier on Endodontically Treated Teeth: A Systematic Review and Meta-Analysis of In Vitro Studies

**DOI:** 10.1155/2022/2789073

**Published:** 2022-01-20

**Authors:** Lucas Peixoto de Araújo, Wellington Luiz de Oliveira da Rosa, Tiago Schlindvein de Araujo, Felipe Immich, Adriana Fernandes da Silva, Evando Piva

**Affiliations:** ^1^Department of Restorative Dentistry, Division of Endodontics, Piracicaba Dental School, State University of Campinas (UNICAMP), Piracicaba, SP, Brazil; ^2^Graduate Program in Dentistry, School of Dentistry, Federal University of Pelotas (UFPEL), Pelotas, RS, Brazil

## Abstract

The main cause of unsuccess in endodontically treated teeth (ETT) is due to bacterial recontamination. The placement of an intraorifice barrier (IOB) has been proposed for preventing this event in cases that the restoration is in an inadequate condition, enhancing the possibilities for predictable long-term success in endodontic therapy. *Objectives*. To evaluate through a systematic review and meta-analysis if it would be necessary to place an IOB in ETT. *Materials and Methods*. The present review is in accordance with the PRISMA 2020 Statement and is registered in the Open Science Framework. Two blinded reviewers carried out a comprehensive search in four databases up to July 10^th^, 2021: MEDLINE, Scopus, Embase, and Web of Science. Eligible studies were the ones which evaluated the use of an IOB in ETT in reducing microleakage with any material of choice and with any methods employed. Only in vitro studies published in English were included. *Results*. A total of thirty in vitro studies were included in the qualitative synthesis, and seven of those were included in the quantitative analyses evaluating the following materials: bioceramic cement, glass-ionomer cement (GIC), and resin-based composite (RBC). Most of the included studies placed an IOB at a 3 mm depth. Reduction in microleakage was observed when an IOB was placed, regardless of the material employed (*p* ≤ 0.01). Among the materials, GIC and RBC performed similarly (*p* > 0.05), with the bioceramic subgroup being statistically superior to the GIC subgroup (*p* ≤ 0.05). *Conclusions*. Although well-designed randomized clinical trials are required, the placement of an intraorifice barrier can significantly reduce microleakage in endodontically treated teeth, and the use of bioceramics as IOB seems to be the best available material for this purpose.

## 1. Introduction

The conventional endodontic treatment has the root canal system disinfection with adequate sealing of the endodontically treated teeth as its final objective. An adequate coronal restoration prevents postoperative reinfection [1, 2], and several studies reported its essential role in the periapical radiolucency healing [2–5]. However, microbiologic contamination can lead to the endodontically treated teeth failure through faults in the sealing ability of the temporary or definitive restoration [6]. Furthermore, resin-based composites placed on teeth can fail in up to 12.4% of the cases [7], and Class II restorations have a relative risk of failure of 2.8 against Class I, and this risk is even higher when more surfaces are involved [8] and if the tooth is endodontically treated [9]. Failure of adhesive restorations due to caries development, fracture, or marginal infiltration is of significant concern since it is one of the major factors related to the survival of endodontically treated teeth [2, 9, 10]. Thus, the use of an intraorifice barrier (IOB) was primarily suggested by Roghanizad and Jones [11] with the purpose of preventing bacterial contamination in cases that the restoration is in an inadequate condition, enhancing the possibilities for predictable long-term success in endodontic therapy.

The technique consists of removing approximately 3.00 mm of the coronal gutta-percha immediately after finishing the root canal obturation and filling the resulting space with a restorative material. Several materials have been described in the literature as options for this technique, and the most commonly reported are glass-ionomer cement (GIC), resin-based composite (RBC), bioceramic cements, or zinc phosphate cements. The ideal characteristics of a material to be used as an intraorifice barrier must be easy to handle, with adhesion to the dental structure, preventing bacterial contamination, to be distinguishable from the natural tooth, and which does not interfere with the final restoration adhesion [12].

Since most of the evidence on this topic is based on in vitro studies, the question still remains whether the clinicians should consider placing an intraorifice barrier and which material is the best for this purpose. Nevertheless, even though microleakage studies can not properly simulate the oral environment, positive laboratory results on reducing microleakage can be expected to perform similarly on adequate clinical conditions [13]. Hence, the main objective of this study was to evaluate through a systematic review and meta-analysis the efficacy of different materials as an intraorifice barrier on coronal microleakage of endodontically treated teeth. The hypothesis tested was if a significant difference would be detected on microleakage of endodontically treated teeth with intraorifice barrier placed when compared with the positive control group without the barrier.

## 2. Methods

### 2.1. Registration and Research Question

The current systematic review is reported complying with the Preferred Reporting Items for Systematic Review and Meta-Analysis (PRISMA 2020) guidelines [14]. Due to the study design nature, the protocol was registered in the Open Science Framework and is available at the following link (osf.io/qxfhy).

### 2.2. Eligibility Criteria

Eligible studies were the ones which evaluated the use of an intraorifice barrier in endodontically treated human teeth in reducing microleakage as the primary outcome with any material of choice and with any methods employed. Only in vitro studies published in English were included.

The exclusion criteria were studies that evaluated the use of an intraorifice barrier during intracoronary bleaching and when utilized as a barrier for post space preparation. Also, in vivo studies were excluded to assure a homogeneity in the methods (samples and outcomes evaluated) of included studies.

### 2.3. Search Strategy

A comprehensive search was carried out up to July 10^th^ of 2021 on the following databases: PubMed/MEDLINE, SciVerse Scopus, Embase, and Web of Science. The search strategy was based on the population-intervention-comparison-outcome (PICO) strategy and aimed at answering the following research question: is the placement of an intraorifice barrier (I) able to prevent microbial microleakage (O) on endodontically treated teeth (P) when compared to teeth filled with gutta-percha and sealer alone (C)?

The specific terms used for the database search were chosen based on the MEDLINE MeSH terms, and it was adapted for the other databases (Table 1). The studies screened had no limit to the published year. After the identification of these articles, they were imported into Mendeley software (Elsevier, Amsterdam, NE) to remove duplicates. Additionally, the pool of studies was improved by searching the references cited by the included studies, and those were hand examined for any further eligible study.

### 2.4. Selection Process

All articles initially found by the search strategy were screened by title and abstract by two blinded and independent reviewers (LPA and FI) utilizing the web application Rayyan (Qatar Computing Research Institute, Doha, QA) [15]. The articles that clearly met the eligibility criteria and those that were uncertain were selected for full-text analysis. The initial interobserver agreement between the two examiners was calculated by Cohen's kappa coefficient (*κ* = 0.89). The papers that met all the eligibility criteria were included in this systematic review, and those which had any disagreement between the two reviewers were clarified through discussion with a third reviewer (WLOR).

### 2.5. Data Collection Process

Data of interest from the included studies were tabulated and interpreted by two independent reviewers (LPA and FI) in an Excel spreadsheet (Microsoft Corporation, Redmond, WA, USA), and another reviewer (TSA) double-checked it. In case of any missing information, the corresponding author of the included study was contacted via e-mail to retrieve any missing data. If the authors did not receive any answer in two weeks, then a second e-mail was sent.

### 2.6. Study Risk of Bias Assessment

Each selected study was assessed for the methodological risk of bias using the revised Cochrane risk of bias tool. This tool was carefully modified according to an adaptation made from a previous systematic review of in vitro studies [16]. Two revisors (LPA and FI) evaluated independently the risk of bias utilizing the following parameters: (1) sample size calculation, (2) samples with similar dimensions, (3) sample teeth examined under a light stereomicroscope, (4) standardization of instrumentation, obturation, and intraorifice barrier space preparation, (5) comprehensible reporting of the study design, [6] samples randomly allocated, (7) presence of a positive and a negative control group, and (8) statistical analysis carried out.

### 2.7. Statistical Analysis

The meta-analyses were performed using Review Manager software version 5.4 (The Nordic Cochrane Centre, The Cochrane Collaboration; Copenhagen, Denmark). Initially, the global analysis was carried out using a random-effects method, and the pooled effect estimates were obtained by comparing the microleakage means from each material used as an IOB and the positive controls (no material used as a barrier). Subgroup analyses were performed considering each material: GIC, MTA, and RBC. Additionally, a comparison among the materials was performed as follows: GIC vs. RBC, GIC vs. MTA, and RBC vs. MTA. Statistical significance was defined as a *p* value ≤ 0.05 (*Z* test), and the statistical heterogeneity among studies was assessed using Cochran's *Q* test, with a threshold *p* value of 0.1, and the inconsistency test (*I*^2^), in which values higher than 75% were considered indicative of considerable heterogeneity [17].

## 3. Results

### 3.1. Search Strategy

**Figure 1:**
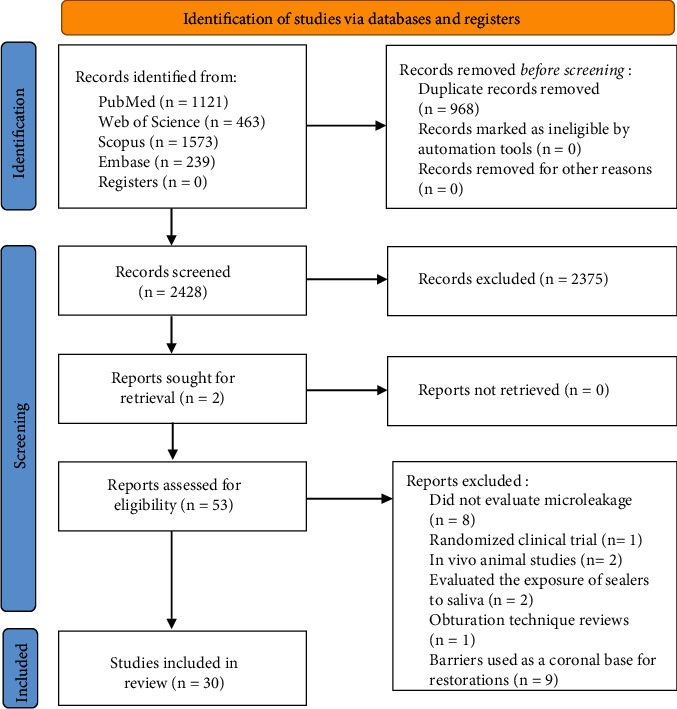
Search flowchart according to the PRISMA 2020 Statement.

The electronic search yielded 3396 potentially relevant records.  Figure 1 is a flowchart that summarizes the article selection process according to the PRISMA 2020 Statement [14]. After removing the duplicates, 2428 articles were screened by titles and abstracts utilizing the web application Rayyan (Qatar Computing Research Institute); 2375 studies were excluded because they did not meet the inclusion criteria, and 53 were held on to full-text analysis. Of these 53 studies, 23 (44%) were not included; of these, 5 evaluated physicochemical characteristics rather than microleakage; 1 was a randomized clinical trial; 2 were in vivo studies; 1 was a review related to obturation techniques; 2 evaluated the exposure of root canal sealers to human saliva; 9 did not use the barriers as IOB but as a coronal base for restorations, and 3 assessed the force required to fracture tooth with intraorifice barriers. The remaining 30 (56%) studies fulfilled all the inclusion criteria and were included in this review.

### 3.2. Descriptive Analyses

**Figure 2:**
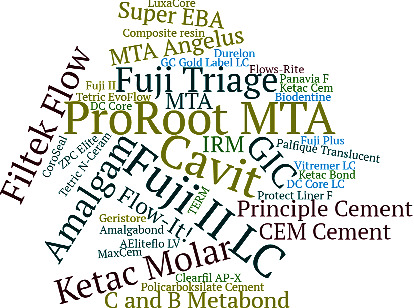
Word cloud representing the materials used as IOBs. Larger font means the materials were used with a greater frequency.

Forty-six different materials were evaluated as an intraorifice barrier in this review, as described in Table 2. Of these, fifteen studies evaluated different types of bioceramic materials [18–32], including 11 ProRoot MTA (Dentsply Sirona, York, PA, USA), 2 MTA Angelus (Angelus, Londrina, PR, BR), 1 EndoCem ZR (Maruchi, Wonju, GO, KR), 2 Biodentine (Septodont, Saint-Maur-des-Fossés, FR), and 2 calcium-enriched mixture (Bionique Dent, Tehran, IR). Thirteen studies [10, 16, 19, 21–25, 27, 29–38] evaluated seven different types of glass-ionomer cements and another eight types of resin-modified glass-ionomer cements, six studies [11, 25, 31, 39–41] evaluated Cavit (3 M ESPE), one [11] evaluated the T.E.R.M (Dentsply Sirona) temporary restorative material, three [11, 21, 22] evaluated the Amalgam (Dentsply Sirona), 5 studies [25, 26, 32, 42, 43] evaluated different types of resin-based composites, and 10 [18, 22, 30, 31, 33–35, 39, 43, 44] evaluated different types of flowable RBCs. One study [34] evaluated a self-etch, resin-based material CoroSeal (Ivoclar Vivadent), one [20] evaluated a zinc phosphate cement, ZPC Elite (GC America), two [40, 43] evaluated the IRM (Dentsply Sirona), other two studies [33, 40] evaluated the Super EBA (Bosworth Company, IL, USA), two [38, 43] evaluated the C&B Metabond (Parkell, Brentwood, NY, USA) with polymethyl methacrylate powder, and one [43] evaluated the Amalgambond Plus (Parkell) also with polymethyl methacrylate powder. Also, other seven studies [20, 29, 33–35, 38, 42] evaluated different types of luting agents, which were 1 LuxaCore (DMG, Hamburg, DE), 1 DC Core LC (Kuraray), 1 DC Core chemically cured (Kuraray), 1 Panavia F (Kuraray), 1 MaxCem (KaVo Kerr, Biberach, DE), 2 principle cement (Dentsply Sirona), 1 Durelon (3M ESPE), and 1 Polycarboxylate cement.  Figure 2 summarizes the materials used in the included studies.

Among the included studies, the sample groups ranged from 30 teeth to 188 teeth with a total of 2111 teeth in all the studies and a mean of 70 teeth per study. There is a predominance in the study samples of single-rooted teeth (25 out of 30 studies, 83%), and the depth of the intraorifice barriers ranged from 1 mm to 4 mm, with the majority of the studies evaluating the materials in a 3 mm depth (16 out of 30 studies, 53%).

Different methodologies to assess microleakage were used (Table 3). Thirteen studies evaluated microleakage by dye penetrant inspection with different types of inks (43,3-%), four studies evaluated by human saliva penetration (13,3-%), six studies evaluated by microbial penetration (20%), other six studies evaluated by a fluid filtration method (20%), and one evaluated the microleakage through a glucose penetration model (3,3-%). The main results of each study are described in Table 2.

### 3.3. Quantitative Analyses

**Figure 3:**
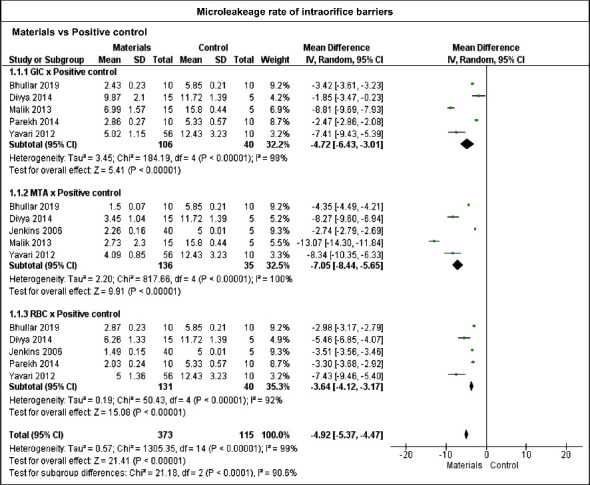
Results for the microleakage analysis of different materials against the positive control groups using a random-effects model. All the materials used as IOBs were significantly different from the positive controls (*p* ≤ 0.05).

Meta-analysis was performed with data sets of microleakage from 7 studies, considering the studies that evaluated microleakage through dye penetrant inspection methods. The global analysis using a random-effects model (Figure 3) demonstrated that the use of an IOB had a statistically lower microleakage rate than the control groups (-4.92 mm, *p* ≤ 0.01). Subgroup analysis considering each material versus control also demonstrated that GICs, MTA, and RBC presented statistically less microleakage rate than the control groups.

**Figure 4:**
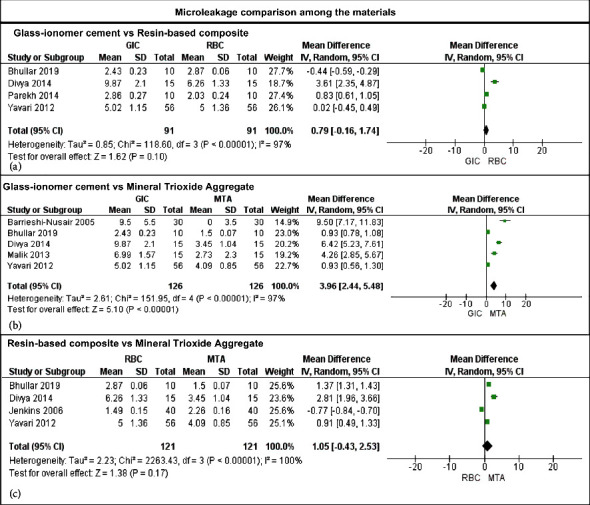
Summary of meta-analysis findings comparing glass-ionomer cement, resin-based composite, and mineral trioxide aggregate against each other using a random-effects model.

The comparison among the materials showed that an intraorifice barrier with RBC showed no statistically different microleakage than GIC (*p* ≥ 0.05), and Cochran's values *Q* and *I*^2^ test were *p* ≤ 0.01 and 97% (Figure 4(a)). Also, MTA promoted a lower and statistically different microleakage than GIC (*p* ≤ 0.01), and the values of Cochran's *Q* test and *I*^2^ were *p* ≤ 0.01 and 97% (Figure 4(b)). Finally, in the comparison between RBC and MTA (Figure 4(c)), no differences were demonstrated between those groups (*p* = 0.17, *I*^2^ = 100%).

### 3.4. Quality Assessment

**Figure 5:**
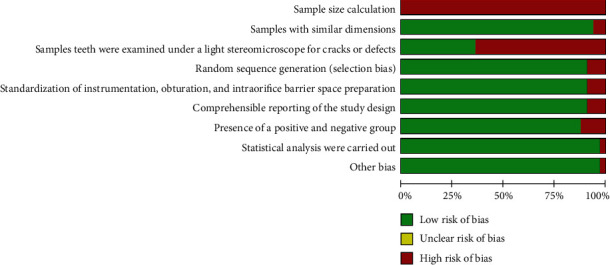
Review authors' judgments about each risk of bias item for each included in vitro study.

According to the parameters established for the quality assessment of the included in vitro studies, of the 30 studies included in this analysis, all the studies scored poorly for the item “sample size calculation” and in 21 of them [10, 15, 16, 18–22, 24, 25, 27, 29, 30, 32, 33, 35, 37–39, 42, 45], a high risk of bias was observed for the item “sample teeth were examined under a light stereomicroscope for cracks or defects.” In contrast, a low risk of bias was detected in the reminiscent parameter evaluated, as shown in Figure 5.

## 4. Discussion

The present systematic review evaluated the efficacy of different materials used as intraorifice barriers to reduce coronal microleakage in endodontically treated teeth. All of the materials tested were statistically superior when compared to the gutta-percha and sealer alone; however, none of the studies showed that any material was capable of entirely preventing microleakage, only to diminish it. The results of our review demonstrated that the placement of an intraorifice barrier at a 3 mm depth into the root canal obturation could improve its sealing ability, providing a more considerable period of time to maintain an adequate coronal sealing. The depth of the barrier seems to be an important factor in reducing microleakage, since some studies compared different intraorifice barrier depths, ranging from 1 mm to 4 mm, and usually, when it was placed at a 3 mm depth, it had better results than when placed at 1 or 2 mm. Additionally, a 3 mm intraorifice barrier depth was performed similarly when placed at a 4 mm depth [11, 28, 33, 37, 44].

Some factors must be taken into consideration in the obtained results regarding methodological limitations of included studies. One of them is the degree of scientific evidence obtained by the in vitro studies that can not properly simulate the clinical oral environment, including the oral microflora synergism, salivary pH, and masticatory stress. In the meta-analysis, it was only possible to analyze data from in vitro studies that evaluated microleakage by dye penetrant inspection with thermocycling with different inks used to assess microleakage, namely, methylene blue, rhodamine-b, India ink, and Pelikan ink. Although it is easy to perform and sophisticated equipment is not requested, it is a limited methodology to assess the real deepest dye penetration point that may result in an underestimation of leakage [46]. Even the bacterial colonization methodologies used to assess microleakage have their own set of limitations because these types of experiments need histological validation [47]. However, in the present review, it was possible to observe the similarity of findings between the in vitro studies and the in vivo studies that assessed histological findings of the effects of intraorifice barriers on periapical inflammation in dogs [48, 49]. In one of them [49], it was observed that the experimental group with an intraorifice barrier had 38% of the roots with periapical inflammation against 89% of the control group with gutta-percha and sealer alone; in the other study [48], no significantly different outcome was observed.

Different materials were tested as intraorifice barriers; the most frequently tested included bioceramics, glass-ionomer cements, resin-based composites, zinc phosphate cements, and other temporary and definitive restorative materials. The use of bioceramics in endodontics is widely appraised for its optimum characteristics regarding biocompatibility, osteoinductive capacity, ability to achieve an excellent hermetic seal due to its hygroscopic expansion capacity, forming a chemical bond with the tooth structure, antibacterial proprieties, and good radiopacity [50–52]. The early MTA generations did not have the ideal characteristics proposed for intraorifice barriers: it had discoloration potential, and it was hard to handle, demanding extra efforts to place it. However, with the recent developments in the bioceramic types of cement, those drawbacks were overcome [53] by replacing the bismuth oxide radiopacifier with zirconium oxide or calcium tungstate, which do not cause tooth discoloration [54, 55], and the handling properties were improved with the introduction of premixed bioceramics, providing a more homogenous mixture and a putty-like consistency that only sets on an appropriate environment [56]. Although none of the included studies that evaluated bioceramic materials as intraorifice barriers used those novel formulations, they may be expected to be easier to handle and place.

Resin-based composites are also of daily use in endodontics for restorative procedures. It was suggested as a proper material as an intraorifice barrier due to its excellent bond properties to tooth tissues and the wide range of color palette to differentiate from the tooth color. Still, the major concern is with the polymerization shrinkage that can lead to marginal microgaps in the barrier interface, compromising the orifice seal. Flowable resin composites are also regarded as a suitable choice for an intraorifice barrier material for their better adaptation to the internal dentin walls; however, the polymerization shrinkage can be higher than the conventional resin-based composites due to their reduced filler, which allows it to have a low viscosity [57]. Another limitation of the included studies in this systematic review is that none of them evaluated the barrier with bulk-fill RBCs, which are well-described in the literature to have a reduced volumetric polymerization shrinkage and stress levels [58] and could potentially leak in a lower intensity than an intraorifice barrier. It is also essential to note that RBCs may have their polymerization process interfered with when in contact with eugenol-based sealers; instead, an epoxy-resin sealer is preferred when placing intraorifice barriers with RBCs [59] as observed in a few studies [34, 39]. Higher concentrations of sodium hypochlorite used to irrigate the root canal system can also impact the sealing ability of intraorifice barriers with RBC because it affects the collagen organization in the dentin extracellular matrix, which are crucial to adhesive systems performed adequately [60, 61]. Moreover, residual NaOCl breaks into sodium chloride and oxygen; the last one has the potential to inhibit the adhesive material polymerization [62]. Meanwhile, it has been shown that chlorhexidine gluconate has no adverse effects on immediate composite-adhesive bonds in dentin or enamel; it even has been reported that endodontic irrigation with chlorhexidine solution significantly increased the shear bond strength to root dentin; although this mechanism is not completely understood yet, it is suggested that the chlorhexidine adsorption by dentin may favor the resin infiltration into dentinal tubules [63–65].

Another issue to be considered when using RBCs is that most adhesive systems have acetone in the formulation. Previous studies reported that acetone-based adhesives do not polymerize well on top of gutta-percha because some components from the gutta-percha can interact with it, and this leaching can inhibit the polymerization process [35, 66]. Although this information seems to be irrelevant to bond coronal restorations, it is an important finding when placing intraorifice barriers because at least 1/3 of the structure to be bonded is coronal gutta-percha.

Even though bioceramics and resin-based composites are entirely different materials with different properties and the meta-analysis in this study showed a high heterogeneity between the included studies, the MTA subgroup was statistically similar to RBCs when used as an intraorifice barrier. However, it seems that the bioceramics have some advantages against RBCs since they are easily removed with ultrasonic tips and represent less danger of procedure errors when removing them, like root perforation or ledge formation [67]. Also, in contrast with RBCs, they have no polymerization shrinkage effect, but they have a hygroscopic expansion [68], which can potentially benefit the marginal intraorifice barrier sealability.

Glass-ionomer cements also have most of the ideal characteristics initially proposed for IOBs [12]. It is a self-adhesive material with satisfactory chemical bonding with root dentine [69], biocompatibility, thermal expansion coefficient close to teeth, and antibacterial activity mainly due to its low pH and fluoride ion release [70]. Another option to be considered is the resin-modified glass-ionomer cement that can be easier to place, and its antibacterial activity is also associated with the light-curing by the release of benzine bromine and benzine iodine. One randomized clinical trial [71] evaluated the outcomes of primary root canal treatment using glass-ionomer cement as an intraorifice barrier for twelve months, and no difference was observed in periapical healing of apical periodontitis; however, it is feasible to say that the follow-up time of twelve months is insufficient to observe expressive failures in dental restorations [72], and thus, endodontic treatment failure due to the lack of an intraorifice barrier providing an additional seal could not be investigated in this timeframe. In the metanalysis, the glass-ionomer cement was able to reduce microleakage when compared to the control group with no barriers. However, when compared to MTA, GICs demonstrated the worst performance in reducing microleakage than other materials.

Although the included studies showed high heterogeneity among the materials tested and methodologies used to evaluate microleakage, the present findings demonstrated that the placement of an intraorifice barrier can improve the coronal seal of the root canals. Future laboratory evidence should explore the benefits of novel sealing materials like flowable bulk-fill composites and premixed bioceramics; also, clinical trials evaluating the effects of intraorifice barriers should be performed with long-term follow-up periods in order to evaluate the intracoronary sealing ability of IOBs during the restorative cycles of rehabilitated teeth. Furthermore, based on the results of the meta-analysis of this study, a better seal can be achieved when bioceramics are used as intraorifice barriers on endodontically treated teeth.

## 5. Conclusion

In spite of the fact that well-designed randomized clinical trials are required, the in vitro results showed that the placement of an intraorifice barrier can significantly reduce microleakage in endodontically treated teeth, and the use of bioceramics as intraorifice barriers seems to be the best available material for this purpose. The results of this study should be carefully interpreted since a high heterogeneity was observed among the studies, and the complexity of interpretation on microleakage findings should be taken into consideration. A call for action to carry out more extensive and long-term clinical studies regarding the placement of intraorifice barriers is desired to clinically understand the advantages of this technique.

## Figures and Tables

**Table 1:** Search strategies.

	Search terms
*PubMed*	
#3	Search #1 AND #2
#2	Search (Coronal Microleakage) OR (Coronal Sealing) OR (Coronal Seal) OR (Coronal Barrier) OR (Intra-coronal Barrier) OR (Intracoronal Barrier) OR (Intraorifice Barrier) OR (Intra-orifice Barrier) OR (Intraorifice Seal) OR (Intra-orifice Seal) OR (Orifice Seal) OR (Orifice Barrier) OR (Intracanal Barrier) OR (Intra-canal Barrier) OR (Intracanal Sealing) OR (Intra-canal Sealing) OR (Barrier Materials) OR (Cervical Barrier)
#1	Search (Tooth, Nonvital) OR (Tooth, nonvital) OR (Nonvital Tooth) OR (Tooth, Devitalized) OR (Devitalized Tooth) OR (Tooth, Pulpless) OR (Pulpless Tooth) OR (Teeth, Pulpless) OR (Pulpless Teeth) OR (Teeth, Devitalized) OR (Devitalized Teeth) OR (Teeth, Nonvital) OR (Nonvital Teeth) OR (Teeth, Endodontically-Treated) OR (Endodontically-Treated Teeth) OR (Teeth, Endodontically Treated) OR (Tooth, Endodontically-Treated) OR (Endodontically-Treated Tooth) OR (Tooth, Endodontically Treated) OR (Root Canal Therapy) OR (Canal Therapies, Root) OR (Canal Therapy, Root) OR (Root Canal Therapies) OR (Therapies, Root Canal) OR (Therapy, Root Canal) OR (Endodontics) OR (Endodontics) OR (Endodontology)
*Embase*	
#3	Search #1 AND #2
#2	Seach “Coronal Microleakage” OR “Coronal Sealing” OR “Coronal Seal” OR “Coronal Barrier” OR “Intra-coronal Barrier” OR “Intracoronal Barrier” OR “Intraorifice Barrier” OR “Intra-orifice Barrier” OR “Intraorifice Seal” OR “Intra-orifice Seal” OR “Orifice Seal” OR “Orifice Barrier” OR “Intracanal Barrier” OR “Intra-canal Barrier” OR “Intracanal Sealing” OR “Intra-canal Sealing” OR “Barrier Materials” OR “Cervical Barrier”
#1	Search “Tooth, Nonvital” OR “Tooth, nonvital” OR “Nonvital Tooth” OR “Tooth, Devitalized” OR “Devitalized Tooth” OR “Tooth, Pulpless” OR “Pulpless Tooth” OR “Teeth, Pulpless” OR “Pulpless Teeth” OR “Teeth, Devitalized” OR “Devitalized Teeth” OR “Teeth, Nonvital” OR “Nonvital Teeth” OR “Teeth, Endodontically-Treated” OR “Endodontically-Treated Teeth” OR “Teeth, Endodontically Treated” OR “Tooth, Endodontically-Treated” OR “Endodontically-Treated Tooth” OR “Tooth, Endodontically Treated” OR “Root Canal Therapy” OR “Canal Therapies, Root” OR “Canal Therapy, Root” OR “Root Canal Therapies” OR “Therapies, Root Canal” OR “Therapy, Root Canal” OR “Endodontics” OR “Endodontics” OR “Endodontology”
*Web of Science*	
#3	Search #1 AND #2
#2	TS=((Coronal Microleakage) OR (Coronal Sealing) OR (Coronal Seal) OR (Coronal Barrier) OR (Intra-coronal Barrier) OR (Intracoronal Barrier) OR (Intraorifice Barrier) OR (Intra-orifice Barrier) OR (Intraorifice Seal) OR (Intra-orifice Seal) OR (Orifice Seal) OR (Orifice Barrier) OR (Intracanal Barrier) OR (Intra-canal Barrier) OR (Intracanal Sealing) OR (Intra-canal Sealing) OR (Barrier Materials) OR (Cervical Barrier))
#1	TS=((Tooth, Nonvital) OR (Tooth, nonvital) OR (Nonvital Tooth) OR (Tooth, Devitalized) OR (Devitalized Tooth) OR (Tooth, Pulpless) OR (Pulpless Tooth) OR (Teeth, Pulpless) OR (Pulpless Teeth) OR (Teeth, Devitalized) OR (Devitalized Teeth) OR (Teeth, Nonvital) OR (Nonvital Teeth) OR (Teeth, Endodontically-Treated) OR (Endodontically-Treated Teeth) OR (Teeth, Endodontically Treated) OR (Tooth, Endodontically-Treated) OR (Endodontically-Treated Tooth) OR (Tooth, Endodontically Treated) OR (Root Canal Therapy) OR (Canal Therapies, Root) OR (Canal Therapy, Root) OR (Root Canal Therapies) OR (Therapies, Root Canal) OR (Therapy, Root Canal) OR (Endodontics) OR (Endodontics) OR (Endodontology))
*SciVerse Scopus*	
#3	Search #1 AND #2
#2	ALL (“Coronal Microleakage”) OR (“Coronal Sealing”) OR (“Coronal Seal”) OR (“Coronal Barrier”) OR (“Intra-coronal Barrier”) OR (“Intracoronal Barrier”) OR (“Intraorifice Barrier”) OR (“Intra-orifice Barrier”) OR (“Intraorifice Seal”) OR (“Intra-orifice Seal”) OR (“Orifice Seal”) OR (“Orifice Barrier”) OR (“Intracanal Barrier”) OR (“Intra-canal Barrier”) OR (“Intracanal Sealing”) OR (“Intra-canal Sealing”) OR (“Barrier Materials”) OR (“Cervical Barrier”)
#1	ALL (“Tooth, Nonvital”) OR (“Tooth, nonvital”) OR (“Nonvital Tooth”) OR (“Tooth, Devitalized”) OR (“Devitalized Tooth”) OR (“Tooth, Pulpless”) OR (“Pulpless Tooth”) OR (“Teeth, Pulpless”) OR (“Pulpless Teeth”) OR (“Teeth, Devitalized”) OR (“Devitalized Teeth”) OR (“Teeth, Nonvital”) OR (“Nonvital Teeth”) OR (“Teeth, Endodontically-Treated”) OR (“Endodontically-Treated Teeth”) OR (“Teeth, Endodontically Treated”) OR (“Tooth, Endodontically-Treated”) OR (“Endodontically-Treated Tooth”) OR (“Tooth, Endodontically Treated”) or (“Root Canal Therapy”) OR (“Canal Therapies, Root”) OR (“Canal Therapy, Root”) OR (“Root Canal Therapies”) OR (“Therapies, Root Canal”) OR (“Therapy, Root Canal”) OR (“Endodontics”) OR (“Endodontics”) OR (“Endodontology”)

**Table 2:** Main results of the included studies.

Study	Experimental groups	Intraorifice barrier depth	Control groups	Main results of the included studies
Roghanizad	Cavit (3M ESPE), TERM (Dentsply), Amalgam (Dentsply)	3 mm	5 positive (no barrier) and 5 negative controls (nail varnish and sticky wax)	A 3 mm intraorifice barrier of Amalgam prevented leakage in 96.4% of the cases, and it was significantly better than Cavit and TERM.
Yavari	Flow-It (Pentron), GC Gold Label LC (GC America), ProRoot MTA (Dentsply)	3 mm	10 positive (no barrier) and 10 negative controls (nail varnish and sticky wax)	A 3 mm intraorifice barrier of ProRoot MTA was statistically superior to GIC or composite resin to minimize recontamination of the remaining gutta-percha.
Malik	Fuji II GIC (GC America), ProRoot MTA (Dentsply)	4 mm	5 positive (no barrier) and 5 negative controls (nail varnish and sticky wax)	A 4 mm intracanal plug of ProRoot MTA exhibited a lower mean leakage than Fuji II GIC, and it may be used to minimize microleakage in endodontically treated teeth.
Lee	ProRoot MTA (Dentsply), EndoCem Zr (Maruchi), MTA Angelus (Angelus), LuxaCore (DMG), Fuji II LC (GC America), ZPC Elite (GC America)	3 mm	5 positive (no barrier) and 5 negative controls (nail varnish)	All the materials allowed infiltration of dye. However, a 3 mm intraorifice barrier of ProRoot MTA showed significantly smaller penetration and less variation than the other materials.
Alikhani	Fuji II LC (GC America)	1, 2, and 3 mm	None	The findings indicated that a 3 mm depth of Fuji II LC intraorifice barrier showed the highest preventive effect on coronal microleakage in endodontically treated teeth.
Shindo	Protect Liner F (Kuraray), Panavia F (Kuraray), DC Core light-cured (Kuraray), DC Core chemically cured (Kuraray), Super EBA (Bosworth), Ketac (3M ESPE)	4 mm	5 positive (no barrier) and 5 negative controls (nail varnish)	A 4 mm intraorifice barrier of Panavia Liner F and Panavia F had the highest sealing ability than the other materials.
Parekh	Fuji II LC (GC America), Tetric N-Flow (Ivoclar Vivadent), Fuji II LC+Tetric N-Flow	3.5 mm	5 positive controls (no barrier)	Tetric N-Flow has shown more leakage than Fuji II LC+Tetric N-Flow and Fuji II LC groups when used as intraorifice barriers.
Bhullar	Biodentine (Septodont), Cention N (Ivoclar Vivadent), Fuji IX GIC (GC America)	3 mm	10 positive (no barrier) and 10 negative controls (nail varnish)	The present study concluded that intraorifice barrier placement provides a better coronal seal and prevents microleakage. Biodentine placed at a 3 mm depth was statistically superior to the other groups.
Pisano	Cavit (3M ESPE), IRM (Dentsply), Super EBA (Bosworth)	3.5 mm	5 positive (no barrier) and 5 negative controls (nail varnish)	A 3.5 mm intraorifice barrier of Cavit leaked the least when compared to the other included materials.
Zakizadeh	Amalgam, Fuji Plus LC (GC America), Geristore (DenMat), ProRoot MTA (Dentsply)	2 mm	5 positive (no barrier) and 5 negative controls (sticky wax)	A 2 mm intraorifice barrier of Fuji Plus might be an effective barrier against saliva contamination for a limited time.
Yavari	ProRoot MTA (Dentsply), Amalgam, Filtek Flow (3M ESPE), CEM cement (BioniqueDent)	3 mm	5 positive (no barrier) and 5 negative controls (nail varnish)	A 2 mm intraorifice barrier of MTA and CEM cement are more effective than Amalgam or composite resin in preventing saliva leakage in endodontically treated teeth.
Tselnik	Gray MTA, white MTA, Fuji II LC (GC America)	3 mm	5 positive (no barrier) and 5 negative controls (epoxy resin)	Intraorifice barriers of MTA and Fuji II LC in a 3 mm depth provided an acceptable coronal seal for up to 90 days in vitro.
Wolcott	Ketac-Bond (3M ESPE), Vitrebond (3M ESPE), trial glass ionomer (GC America)	2 and 3 mm	5 positive (no barrier) and 5 negative controls (epoxy resin)	The intraorifice seal provided by the Vitrebond was significantly better than the seal in teeth without intraorifice barriers (*p* < 0.05).
Barrieshi-Nusair	ProRoot MTA (Dentsply), glass ionomer cement	4 mm	5 positive (no barrier) and 5 negative controls (sticky wax)	Mineral trioxide aggregate, when placed coronally in 4 mm thickness over gutta-percha, seals the canal content significantly more than glass ionomer does.
Jenkins	Cavit (3M ESPE), ProRoot MTA (Dentsply), Tetric (Ivoclar Vivadent)	1, 2, 3, and 4 mm	5 positive (no barrier) and 5 negative controls (nail varnish)	The results of this study indicated that, at all depths, Tetric demonstrated a significantly better seal than either MTA or Cavit.
Sauáia	Cavit (3M ESPE), Vitremer LC (GC America), Flow-It (Pentron)	3 mm	10 positive (no barrier) and 10 negative controls (nail varnish)	The results showed that Cavit sealed significantly better than Vitremer and Flow-It when used as intraorifice filling materials at a 3 mm depth.
Divya	Composite resin, gray MTA, white MTA, glass ionomer cement	4 mm	5 positive (no barrier) and 5 negative controls (nail varnish)	None of the materials prevented the microleakage completely. However, the groups restored with MTA showed significantly better results in preventing microleakage than the other groups.
Ramezanali	MTA Angelus (Angelus), CEM cement (BioniqueDent), Biodentine (Septodont)	3 mm	5 positive (no barrier) and 5 negative controls (nail varnish)	There were no statistical differences between the experimental groups. However, CEM cement at 3 mm depth exhibited the least microleakage. CEM cement, Biodentine, and MTA effectively provide an efficient seal when used as intraorifice barriers in endodontically treated teeth.
Galvan	Amalgambond Plus with PMMA powder (Parkell), C&B Metabond with PMMA powder (Parkell), Æliteflo LV composite (BISCO), Palfique translucent composite (Tokuyama), IRM (Dentsply)	Pulpal floor and 3 mm intraorifice depth	1 positive (no barrier) and 1 negative control (cyanoacrylate)	All the four adhesive resins effectively decreased coronal microleakage, with Amalgambond producing the best seal at all times. IRM, however, demonstrated extensive leakage at 1 and 3 months.
Wells	Principle cement (Dentsply) and C&B Metabond (Parkell)	Pulpal floor and 2 mm intraorifice depth	1 positive (no barrier) and 1 negative control (nail varnish)	The seal provided by C&B Metabond was superior to the seals produced by principle. However, by 1 week, there were no significant differences among the seals.
Maloney	Fuji Triage (GC America)	1 and 2 mm	5 positive (no barrier) and 5 negative controls (nail varnish)	Teeth with Fuji Triage intracoronal barriers leaked significantly less than teeth without barriers. There was no significant difference between the 1 and 2 mm barriers. However, there was a trend towards less fluid movement when a thicker barrier was placed.
Jack	Resilon and Epiphany (Resilon Research), Fuji Triage (GC America)	2 mm	2 positive (no barrier) and 5 negative controls (nail varnish)	The placement of a 2 mm Triage glass ionomer intraorifice barrier after gutta-percha obturation resulted in significantly more resistance to fluid movement than the other groups.
John	Fuji Triage (GC America), gray MTA, white MTA	2 mm	5 positive (no barrier) and 5 negative controls (nail varnish)	No statistically significant difference in fluid flow leakage was found between the experimental groups. Both Fuji Triage and MTA provide superior intraorifice seal than the control group.
Bayram	CoroSeal (Ivoclar Vivadent), Ketac Molar Easymix (3M ESPE), Filtek Flow (3M ESPE), Polycarboxylate cement	2 mm	5 positive (no barrier) and 5 negative controls (nail varnish)	CoroSeal at a 2 mm intraorifice depth was the most effective material among the other groups in reducing the coronal leakage when compared to flowable composite, fissure sealant, and polycarboxylate cement.
Mohammadi	Gray MTA, white MTA, principle cement (Dentsply)	3 mm	3 positive (no barrier) and 3 negative controls (epoxy resin)	The results indicated that MTA, when placed coronally in 2 mm thickness over gutta-percha, significantly reduced the bacterial penetration.
Fathi	Ketac Cem (3M ESPE), Clearfil AP-X (Kuraray), Maxcem (Kerr)	2 mm	5 positive (no barrier) and 5 negative controls (inoculated with sterile BHI broth)	There was no statistically significant difference in the bacterial penetration of Ketac-Cem, Clearfil Protect Bond/Clearfil AP-X, and Maxcem as intracoronal barriers by 120 days.
Valadares	Cavit (3M ESPE)	2 and 3 mm	25 positive (no barrier) and 5 negative controls (cyanoacrylate)	Applying a 3 mm intraorifice barrier of Cavit practically eliminated the microleakage from E. faecalis in the apical third of the root canal system.
Rashmi	ProRoot MTA (Dentsply), Fuji II LC (GC America), Flows-rite (PulpDent)	3 mm	20 positive (no barrier) and 20 negative controls (epoxy resin)	Based on this study, it can be concluded that 3 mm of Fuji II LC provided a better intraorifice seal than MTA and flowable resin composite.
Celik	Ketac Molar Easymix (3M ESPE), Durelon (3M ESPE), Vitrebond (3M ESPE), Filtek Flow (3M ESPE)	1 mm	15 positive (no barrier) and 5 negative controls (nail varnish)	1 mm intraorifice barrier of Ketac Molar Easymix demonstrated statistically lower leakage than the flowable resin composite group.
Bailón-Sanchéz	ProRoot MTA (Dentsply), Cavit (3M ESPE), Tetric EvoFlow (Ivoclar Vivadent)	4 mm	6 positive (no barrier) and 6 negative controls (nail varnish)	ProRoot MTA, Cavit, and Tetric EvoFlow demonstrated similar leakage values when used as an intraorifice barrier at a 4 mm depth.

**Table 3:** Demographic data of the included studies.

Study	Year	Country	Methodology	Sample size (per group)	Tooth group
Roghanizad	1996	United States	2% methylene blue dye penetration	94 (28)	Maxillary incisors
Yavari	2012	Iran	2% methylene blue dye penetration	188 (56)	Single-rooted premolars
Malik	2013	India	2% methylene blue dye penetration	70 (30)	Single-rooted premolars
Lee	2015	South Korea	1% methylene blue dye penetration	70 (10)	Single-rooted premolars
Alikhani	2020	Iran	2% methylene blue dye penetration	45 (15)	Single-rooted teeth
Shindo	2004	Japan	2% methylene blue dye penetration	100 (15)	Single-rooted teeth
Parekh	2014	India	Rhodamine-B dye penetration	40 (10)	Single-rooted premolars
Bhullar	2019	India	Rhodamine-B dye penetration	50 (10)	Single-rooted teeth
Pisano	1998	United States	Human saliva penetration	74 (20)	Single-rooted teeth
Zakizadeh	2008	United States	Human saliva penetration and micro-CT evaluation	50 (10)	Single-rooted teeth
Yavari	2012	Iran	Human saliva penetration	70 (15)	Single-rooted premolars
Tselnik	2004	United States	Human saliva penetration	78 (18)	Single-rooted teeth
Wolcott	1999	United States	Proteus vulgaris penetration	110 (25)	Single-rooted teeth
Barrieshi-Nusair	2005	Kuwait	Pelikan ink penetration	70 (30)	Single-rooted teeth
Jenkins	2006	United States	India ink penetration	130 (40)	Single-rooted teeth
Sauáia	2006	Brazil	India ink penetration	80 (20)	Maxillary and mandibular molars
Divya	2014	India	India ink penetration	70 (15)	Single-rooted premolars
Ramezanali	2017	Iran	India ink penetration	76 (22)	Single-rooted premolars
Galvan	2002	United States	Fluid filtration model	52 (10)	Mandibular molars
Wells	2002	United States	Fluid filtration model	62 (15)	Maxillary and mandibular molars
Maloney	2005	United States	Fluid filtration model	30 (10)	Single-rooted premolars
Jack	2008	United States	Fluid filtration model	34 (15)	Single-rooted teeth
John	2008	United States	Fluid filtration model	40 (10)	Single-rooted teeth
Bayram	2013	Turkey	Fluid filtration model	50 (10)	Maxillary incisors
Mohammadi	2006	Iran	Enterococcus faecalis penetration	51 (15)	Single-rooted teeth
Fathi	2007	United States	Enterococcus faecalis penetration	55 (15)	Single-rooted teeth
Valadares	2011	Brazil	Enterococcus faecalis penetration	70 (20)	Single-rooted teeth
Rashmi	2018	India	Enterococcus faecalis penetration	100 (20)	Single-rooted teeth
Celik	2006	Turkey	Staphylococcus epidermitis penetration	60 (10)	Single-rooted premolars
Bailón-Sanchéz	2011	Spain	Glucose penetration	42 (10)	Single-rooted teeth

## Data Availability

The registration is available at the Open Science Framework at the following link: https://osf.io/qxfhy/.

## Supplementary Materials

Supplementary Materials PRISMA 2020 checklist.
